# Improvements in the Duckweed-Microbe Co-cultivation Method for the Stable and Efficient Isolation of Rarely Cultivated Bacteria Using Microfilter Membranes

**DOI:** 10.1264/jsme2.ME24075

**Published:** 2025-08-20

**Authors:** Yosuke Morishita, Tomoki Iwashita, Manabu Kanno, Hideyuki Tamaki, Yoichi Kamagata, Tadashi Toyama, Kazuhiro Mori, Masaaki Morikawa, Yasuhiro Tanaka

**Affiliations:** 1 Graduate School of Engineering, University of Yamanashi, 4–3–11 Takeda, Kofu, Yamanashi 400–8511, Japan; 2 Bioproduction Research Institute, AIST, 1–1–1 Higashi, Tsukuba, Ibaraki 305–8566, Japan; 3 Division of Biosphere Science, Graduate School of Environmental Science, Hokkaido University, Kita–10 Nishi–5, Kita–ku, Sapporo 060–0810, Japan; 4 Graduate School of Life and Environmental Sciences, University of Yamanashi, 4–4–37 Takeda, Kofu, Yamanashi 400–8510, Japan

**Keywords:** *Verrucomicrobiota*, duckweed, microbial isolation, filterable microbes

## Abstract

We recently proposed a novel microbial isolation technique, the “duckweed-microbe co-cultivation method”, for isolating a wide variety of microbes, including rarely cultivated microbes. This method involves the inoculation of aseptic duckweed with environmental microbes followed by co-cultivation for a set period. Plants and their surrounding medium are then used as microbial sources for isolation in the conventional agar plate method. In the present study, we improved the method by using microfilter membranes (pore sizes of 0.8–1.2‍ ‍μm) to pretreat microbial inocula, which increased the isolation efficiency of rarely cultivated microbes representing the phylum *Verrucomicrobiota*.

Recent advances in mole­cular-based techniques have revealed the existence of a wide variety of prokaryotes in‍ ‍nature, with 402,709 species (as of Jan. 13, 2024) proposed in the Genome Taxonomy Database (https://gtdb.ecogenomic.org/). Of these, 19,884 valid species with isolates are registered in the List of Prokaryotic Names with Standing in Nomenclature (https://lpsn.dsmz.de/) as of Jun. 13, 2024, which accounts for only 4.9% of the number of species proposed above. This indicates that numerous prokaryotic species are yet-to-be-cultivated or rarely cultivated microbes (Amann *et al.*, 1995; Gans *et al.*, 2005). Some of these microbes are considered to have beneficial functions, such as the ability to produce new antibiotics and degrade persistent toxic chemicals (Stevenson *et al.*, 2004; de Castro *et al.*, 2014; Ling *et al.*, 2015; Danso *et al.*, 2019). Therefore, many attempts have been made to isolate and cultivate these microbes (Bruns *et al.*, 2002; Nichols *et al.*, 2010; Mahler *et al.*, 2021; Seo *et al.*, 2023).

We previously exami­ned microbes inhabiting the roots of the floating aquatic plant, *Spirodela polyrhiza* (duckweed), and four emergent plants, *Phragmites australis* (reed), *Lythrum anceps* (Japanese loosestrife), *Iris pseudacorus* (yellow iris), and *Scirpus juncoides* (tule), and revealed that these aquatic plants harbor taxonomically diverse and novel microbes (Matsuzawa *et al.*, 2010; Tanaka *et al.*, 2012; Tanaka *et al.*, 2017). To support these findings, we proposed the following hypotheses: 1) aquatic plants continuously recruit specific microbial species from the diverse microbial communities present in their surroundings, which may attach and adapt to plants’ root environments. 2) Plants also acclimate these microbes into an easily cultivable state, potentially through the release of root exudates, the composition and roles of which remain unclear. Based on these findings and hypotheses, we propose that the co-cultivation of an aseptic plant and microbes from an environmental sample may yield unique microbial consortia, including a wide variety of taxonomically novel microbes. Among aquatic plants, duckweed was considered the most suitable for constructing the microcosm due to its rapid growth and ease of sterilization. With this background, we recently proposed the “duckweed-microbe co-cultivation method”, in which aseptic duckweed is inoculated with environmental microbes, co-cultivated for a set period, and the resultant plants and their co-cultivated medium are then used as a source for microbial isolation (Tanaka *et al.*, 2018).

A wide variety of microbes, including rarely cultivated bacterial phyla, such as *Armatimonadota* and *Verrucomicrobiota*, which are recalcitrant to conventional cultivation methods, were successfully obtained from the roots of Japanese loosestrife and river water by introducing a co-culture step with duckweed (Tanaka *et al.*, 2018, 2024). We found that microbes belonging to the phylum *Verrucomicrobiota* dominated in the duckweed root and were cultivated on agar medium. Additionally, this phylum was dominant in the co-cultivated medium with a high yield (7.8–13.0%) and its isolation rate from medium samples was up to 22.2% (Tanaka *et al.*, 2024).

On the other hand, Portillo *et al.* (2013) reported that the cell sizes of bacterial phyla known as yet-to-be cultured or‍ ‍rarely cultivated bacteria in soil were generally smaller than those of readily cultivable bacterial phyla, such as *Pseudomonadota*, *Actinomycetota*, and *Bacillota*. This finding prompted us to hypothesize that the microfiltration of microbial inocula (environmental samples) as applied in the duckweed-microbe co-cultivation system may selectively reduce the presence of readily cultivable bacteria in the system, thereby allowing a different microbial community from that in the original environmental sample to grow, favoring rarely cultivated microbes. To confirm this, we constructed a series of duckweed-microbe co-cultivation systems using river water samples that had been pre-treated using microfilters with various pore sizes as microbial inoculants, and then analyzed the microbial communities that formed in these systems. Microbial isolation from the improved duckweed-microbe co-cultivation system was also performed.

Duckweed (*S. polyrhiza*) was aseptically grown in Toyama medium (Toyama *et al.*, 2006) and kept at 25°C in a plant growth chamber (photosynthetic photon flux density [PPFD] of 132‍ ‍μmol m^–2^ s^–1^; 16:8‍ ‍h light-dark cycle).

River water samples collected from three rivers in Kofu city (Yamanashi Prefecture, Japan): Aikawa River, AI_RW (collected on 12 July 2021), Arakawa River, AR_RW, and Fujikawa River, FJ_RW (both collected on 30 August 2021), were used as the microbial sources. River water samples (100‍ ‍mL each) were treated by pressure filtration using 25-mm Isopore membrane filters (Merck) with pore sizes of 10‍ ‍μm (only for the water sample from Aikawa river), 5.0‍ ‍μm, 2.0‍ ‍μm, 1.2‍ ‍μm, and 0.8‍ ‍μm. Filtered (flow-through liquid) and non-filtered samples were used as microbial sources. Twelve plants of aseptic duckweed were transplanted into each microbial source (100‍ ‍mL) for inoculation by microbes and were then kept in a plant growth chamber at 25°C for 1 day. After the inoculation, plants were washed twice with 36‍ ‍mL of sterilized Toyama medium and transferred to a new medium (100‍ ‍mL in a 500-mL plant culture bottle). After 5 days (1st batch), twelve plants were collected from the co-cultivated system, washed with 36‍ ‍mL of sterilized Toyama medium, and transferred to fresh medium in a plant culture bottle. They were then cultivated for 5 days (2nd batch) in the plant growth chamber (10 days of cultivation in total). By using non-filtered and filtered river water samples, 16 duckweed-microbe co-cultivation systems were constructed (Fig. 1). To simplify the experimental series in the present study, we collected only co-cultivated media from systems constructed with the 2nd batch to confirm improvements in the duckweed-microbe co-cultivation method. This approach was selected because the rarely cultivated bacterial group, *Verrucomicrobiota*, was more dominant in the co-cultured medium than on duckweed and, thus, was easily isolated, as stated above (Tanaka *et al.*, 2024).

The collected river water and co-cultivated media from the systems (50‍ ‍mL each) were subjected to a filtration treatment (Isopore Membrane Filters 0.22‍ ‍μm, Merck Millipore) for the ana­lysis of microbial compositions based on 16S rRNA gene amplicons. Total DNA was extracted from filtered samples using the Cica Geneus DNA Extraction Kit (Kanto Chemical) as previously described (Tanaka *et al.*, 2024). Extracted DNA was subsequently purified with Zymo-Spin columns (Zymo Research). 16S rRNA gene fragments (the V4 region) were amplified using the above purified DNA as a template with the primers Eub-515F (5′-ACACTCTTTCCCTACACGACGCTCTTCCGATCT-GTGCCAGCMGCCGCGGTAA-3′; the sequence for 2nd PCR is underlined) and Eub-806R (5′-GTGACTGGAGTTCAGACGTGTGCTCTTCCGATCT-GGACTACHVGGGTWTCTAAT-3′; the sequence for 2nd PCR is underlined). The preparation of 2nd PCR amplicons and sequencing (using the Miseq sequencer, Illumina) was outsourced to Bioengineering Lab. The raw sequence data obtained were imported into QIIME2 (ver. 2022.11.1). After raw sequence data were modified by the method using divisive amplicon denoising algorithm 2 (DADA), forward and reverse reads were merged and classified into amplicon sequence variants (ASVs). ASVs were subjected to taxonomic classification using the SILVA SSU database (ver. 138), and those classified into mitochondria and chloroplasts were removed. Sequence data were deposited in the DNA Data Bank of Japan under the accession number DRA018862. A heat map was created by R (ver. 4.2.3) using the gplots package (3.1.3), and a cluster ana­lysis was performed using the dist function “Canberra” and the Ward method.

Microbial isolation from three river water samples and co-cultivated media, not including the plant body, collected from the above constructed systems was conducted using DTS agar plates as previously described (Matsuzawa *et al.*, 2010). Serially diluted samples were prepared with sterilized Toyama medium, 50‍ ‍μL of each was inoculated on DTS agar plates in triplicate for each sample, and plates were incubated at 25°C under dark conditions for two weeks.

The 16S rRNA genes of isolates were amplified by PCR using the primers Eub-8F (5′-AGAGTTTGATCMTGGCTCAG-3′; Weisburg *et al.*, 1991) and Eub-1512R (5′-ACGGYTACCTTGTTACGACTT-3′; Kane *et al.*, 1993) as described in our previous study (Matsuzawa *et al.*, 2010). Amplified DNAs were subjected to a RFLP ana­lysis using two types of restriction endonucleases *Hha*I and *Hap*II (TaKaRa). 16S rRNA gene fragments from representative isolates of each‍ ‍RFLP group were purified with the Exo-CIP Rapid PCR Cleanup Kit (BioLabs) and sequenced as previously described (Tamaki *et al.*, 2005). Sequence data were compared with the 16S rRNA gene sequences of known bacterial species using the BLAST search program (https://blast.ncbi.nlm.nih.gov). GenBank/EMBL/DDBJ accession numbers for the 16S rRNA gene sequences of isolates are LC822568–LC822651.

A 16S rRNA gene amplicon ana­lysis targeting samples from 16 duckweed-microbe co-cultivation systems (sample IDs are shown in Fig. 1) and their microbial inocula yielded 1,170,623 sequences, which were classified into 308 to 546 ASVs with Good’s coverage of the library ranging from 99.98 to 100% (Table S1). The alpha diversity of each sample was evaluated based on the Shannon index (Table S1). Scores for samples from the duckweed-microbe co-cultivation systems using filtered and non-filtered river water samples as microbial sources ranged from 7.99 to 8.69 and from 7.16 to 8.36, respectively, indicating that the pretreatment of microbial inocula by microfiltration did not affect the abundance of microbial diversity that formed in the system.

At the phylum level, ASVs were classified into 49 taxonomic groups, and 20 were distributed in at least one river water or co-cultivation medium sample at more than 1.0% (Fig. 2). The dominant phyla in non-filtered river water-inoculated systems (AI_NFCM, FJ_NFCM, and AR_NFCM) and their microbial sources (three river water samples; AI_RW, FJ_RW, and AR_RW) were compared to confirm whether the duckweed-microbe co-cultivation method effectively reconstructed the microbial community, as reported in our previous study (Tanaka *et al.*, 2018, 2024). In samples collected from Aikawa and Arakawa rivers, the phylum *Pseudomonadota* was the dominant taxon in samples from the non-filtered river water-inoculated systems (AI_NFCM and AR_NFCM) and the original microbial sources (river water; AI_RW and AR_RW). On the other hand, in samples from Fujikawa river, the phylum *Cyanobacteriota* was dominant in the river water sample (FJ_RW), while the phyla *Pseudomonadota* (36.5%) and *Bacteroidota* (36.6%) were dominant at similar levels in the non-filtered river water-inoculated system (FJ_NFCM). Regarding the second most dominant phyla, *Bacteroidota*, *Planctomycetota*, and *Verrucomicrobiota* were detected in AI_RW, AR_RW, and FJ_RW, respectively. In contrast, in the non-filtered river water-inoculated systems, *Myxococcota* was the second most dominant in AI_NFCM and *Bacteroidota* in AR_NFCM (the second most dominant in FJ_NFCM was stated above). These results indicate that the microbial communities in co-cultivated systems were reconstructed from those in each river water sample using the duckweed-microbe co-cultivation method.

We previously reported that the abundance of the phylum *Verrucomicrobiota* markedly increased and reached 7.8–13.0% in a co-cultivated medium after a duckweed-microbe co-cultivation, whereas its abundance in the original microbial source (river water) was only 1.5% (Tanaka *et al.*, 2024). Therefore, we compared the distribution rates of *Verrucomicrobiota* among river water samples (AI_RW, FJ_RW, and AR_RW) and samples from non-filtered river water-inoculated systems (AI_NFCM, FJ_NFCM, and AR_NFCM). In river water samples, the abundance of *Verrucomicrobiota* was 1.2% for AI_RW, 26.5% for FJ_RW, and 1.8% for AR_RW. In contrast, the scores for samples from the non-filtered river water-inoculated systems, AI_NFCM, FJ_NFCM, and AR_NFCM, were 0.9, 18.9, and 7.7%, respectively. The abundance of *Verrucomicrobiota* did not increase in two of the three systems. The result obtained for the FJ_NFCM system was attributed to the high abundance of the phylum in the original microbial source. In contrast, a high score was still observed in the sample, and the distribution of a taxonomic lineage (the family *Opitutaceae*) within the phylum markedly increased (see below). This suggests that the duckweed-microbe co-cultivation method was also effective for accumulating the microbial group in this sample. However, the sample from the AI_NFCM system showed neither an increase in *Verrucomicrobiota* nor their high abundance, indicating that the effects of the duckweed-microbe co-cultivation method varied depending on the sample.

We then compared microbial compositions between non-filtered and filtered river water-inoculated systems to examine the effects of microfiltration on the microbial inoculum. At the phylum level, *Pseudomonadota* was dominant in all filtration-inoculated systems of Aikawa and Arakawa river water samples, and this result aligned with that of their non-filtered sample-inoculated systems. In Fujikawa river water-related systems, the phylum *Pseudomonadota* was also dominant, except for the system FJ_2.0CM, in which the‍ ‍dominant phylum was *Bacteroidota*. In Aikawa river water-related systems, the abundance of the phylum *Verrucomicrobiota* was higher in all filtered sample-inoculated systems (10.7% for AI_10CM, 1.9% for AI_5.0CM, 10.4% for AI_2.0CM, 28.2% for AI_1.2CM, and 23.1% for AI_0.8CM) than in the non-filtered sample-inoculated system (AI_NFCM; 0.9%) and their original microbial source (AI_RW; 1.2%). On the other hand, the phylum was distributed in co-cultivated media from filtration-inoculated systems derived from Fujikawa and Arakawa river water samples with rates ranging from 11.1 to 19.1% and from 1.9 to 28.2%, respectively, indicating that its high distribution in samples from filtered sample-inoculated systems was maintained or greater than that in non-filtration sample-inoculated systems. These results suggest that pretreating the microbial source with microfilters (although its dependence on pore sizes was unclear) supported the preferential growth and colonization of the phylum *Verrucomicrobiota* in the duckweed-microbe co-cultivation system.

To compare the microbial communities in each sample in more detail, we analyzed data at the family level (Fig. S1). Based on the results obtained, a hierarchical cluster heat map was constructed (Fig. 3). Nineteen samples were divided into two large clusters (clusters 1 and 2), and the original microbial sources, *i.e.*, three river water samples, were all included in the same cluster (cluster 2). In con­trast,‍ ‍cluster 1 formed with samples from duckweed-microbe co-cultivation systems, and was further divided into 4 sub-clusters (clusters 1-1, 1-2, 1-3, and 1-4). Within these sub-clusters, three (clusters 1-1, 1-3, and 1-4) did not include samples from non-filtered river water-inoculated systems. Additionally, when focusing on samples from <1.2‍ ‍μm microfiltration-inoculated systems (AI_1.2CM, FJ_1.2CM, AR_1.2CM, AI_0.8CM, FJ_0.8CM, and AR_0.8CM), none clustered with the original microbial sources or samples from the systems using non-filtered river waters. These results indicate that the pretreatment of microbial inocula for the duckweed-microbe co-cultivation method using microfilter membranes, particularly microfilters with pore sizes of 1.2 and 0.8‍ ‍μm, effectively changed the microbial community in the system.

In addition, most of the phylum *Verrucomicrobiota* detected in duckweed-microbe co-cultivation systems were in the family *Opitutaceae*, covering 63.0 to 99.2% of this‍ ‍phylum. In comparison to the non-filtered sample-inoculated systems, an increase in the abundance of *Opitutaceae* and/or the stabilization of the accumulating effects of this family was observed in the filtered sample-inoculated system (Fig. S1). Since most species belonging to the family *Opitutaceae* are cocci with small cell sizes (ranging between 0.3 and 1.0‍ ‍μm) (Shieh and Jean, 1998; Chin *et al.*, 2001; Rodrigues and Isanapong, 2014; Rochman *et al.*, 2018; Tegtmeier *et al.*, 2018; Wertz *et al.*, 2018; Baek *et al.*, 2019; Pitt *et al.*, 2020; Chen *et al.*, 2020; Nakai, 2020), this result may be attributed to their cell size, which may have allowed them to easily pass through filter pores in the microfiltration pretreatment, resulting in the preferential inoculation of duckweed.

The mole­cular-based ana­lysis showed that microbial communities differed among samples from non-filtered water and filtered water-inoculated systems. Therefore, we conducted microbial isolation and cultivation from these samples to establish whether it was possible to obtain phylogenetically different microbes. Based on the results of the hierarchical cluster ana­lysis of microbial communities (Fig. 3), we targeted samples from Fujikawa river water-related systems that were distributed over all sub-clusters within cluster 1. Since the systems inoculated with samples filtered with pore sizes <1.2‍ ‍μm effectively changed the microbial community, we also targeted the sample from AR_1.2CM, which showed the highest microbial diversity (Shannon index) among all samples (Table S1), as a representative. Additionally, the original microbial sources, FJ_RW and AR_RW, were subjected to microbial isolation as controls.

Table S2 shows the maximum viable counts grown on the plates after 14 days of cultivation. In Fujikawa-related samples, the viable count for the sample from FJ_NFCM was 1.9×10^6^ CFU mL^–1^, while those from the filtered water-inoculated duckweed microbe co-cultivation systems ranged from 3.2×10^6^ CFU mL^–1^ to 1.3×10^7^ CFU mL^–1^. These results suggest that microfilter treatments of microbial sources did not affect the amounts of microbes grown in the systems. A total of 210 strains were isolated and grouped by‍ ‍PCR-RFLP targeting the 16S rRNA gene. As a result, 84‍ ‍RFLP groups were obtained (Table 1). The nucleotide sequence of the 16S rRNA gene from a representative strain of each RFLP group was elucidated, and the data obtained were subjected to a BLAST database search for their taxonomic positions. While direct comparisons of microbial communities across the samples were limited due to the small and variable number of isolates (16 to 38 isolates for each sample), the taxonomic composition at the family level of the isolates in each sample appeared to be distinct, particularly between samples from duckweed-microbe co-cultivation systems and two river waters (Fig. S2). In the samples from co-cultivation systems derived from Fujikawa river water, 22 bacterial families were obtained, 14 of which were only isolated from the filtered sample-inoculated system (Fig. S3A). This result indicates that in contrast to the non-filtered sample-inoculated system, the systems inoculated with filtered microbes enabled taxonomically different types of microbes to be obtained. This was further supported by the hierarchical cluster heat map based on the family level of microbial compositions in isolates (Fig. S3B).

When isolates with a 16S rRNA gene sequence identity <98.7% to that from any known bacterial species were regarded as taxonomically novel microbes, as defined by Stackebrandt and Ebers (2006), four novel microbial strains were obtained from each of the original microbial sources, FJ_RW and AR_RW, and accounted for 28.6 and 25% of all isolates, respectively. On the other hand, in Fujikawa river water-related co-cultivation systems, taxonomically novel isolates were 1 strain for FJ_NFCM (4.3%), 6 for FJ_5.0CM (24%), 15 for FJ_2.0CM (48.4%), 16 for FJ_1.2CM (42.1%), and 18 for FJ_0.8CM (48.6%), indicating that the microfiltration of microbial inocula for the co-cultivation system using a pore size <2.0‍ ‍μm effectively isolated taxonomically novel microbes. A high yield of taxonomically novel microbial isolation was achieved in the sample from AR_1.2CM, with a rate of 65.4% (17 strains), whereas the rate in the river water sample (AR_RW) was 24%, as stated above. Two isolates from the AR_1.2CM sample belonged to the phylum *Armatimonadota* or *Myxococcota*, which are rarely cultivated bacterial lineages (Tamaki *et al.*, 2011; Mohr, 2018) (the strains in RFLP group No. 46 and RFLP group No. 84, respectively).

Additionally, focusing on the phylum *Verrucomicrobiota*, which was stably and frequently distributed in the co-cultivation systems derived from Fujikawa river water samples by the 16S rRNA gene amplicon ana­lysis (ranging from 11.1 to 19.1%), strains within this phylum were only isolated from the systems inoculated with the microbes that passed through microfilters with pore sizes <2.0‍ ‍μm (2 strains from FJ_2.0CM, 8 from FJ_1.2CM, and 2 from FJ_0.8CM). All of these isolates belong to the family *Opitutaceae* (Table 1, Fig. S2), reflecting the results of the mole­cular-based microbial community ana­lysis. Although the reason why *Opitutaceae* strains were not obtained from FJ_NFCM and FJ_5.0CM, which included a high abundance of microbes in the mole­cular-based ana­lysis, remains unclear, it may be attributed to direct and indirect interactions (*e.g.*, competition) between the family and other microbes on the agar plate.

In the case of Arakawa river-related samples, *Verrucomicrobiota* were not isolated from river water samples. However, 9 strains (comprising 34.6% of all isolates), which were all members of the family *Opitutaceae* and also found in the Fujikawa river water-related co-cultivation systems, were successfully isolated from AR_1.2CM. Since the highest isolation yield of the phylum for Fujikawa river water-related samples was observed in the sample from FJ_1.2CM as stated above, the use of a microfilter with a pore size of 1.2‍ ‍μm to pretreat inocula for a duckweed-microbe co-cultivation was considered to be the best approach for improving the method in terms of the stable and efficient isolation of *Verrucomicrobiota* strains.

In conclusion, the present study provides useful information for improving the duckweed-microbe co-cultivation method as follows: (i) the filtration of the microbial source using a microfilter with a pore size <1.2‍ ‍μm markedly changed the microbial community that formed in the system, and (ii) the community stably included microbes belonging to the phylum *Verrucomicrobiota* with a high abundance. Additionally, (iii) the pretreatment using a microfilter with a pore size <2.0‍ ‍μm enabled the isolation of taxonomically novel microbes with a high yield, specifically, the microfilter with a pore size of 1.2‍ ‍μm was highly effective for isolating microbes belonging to the rarely cultivated bacterial groups representing the phylum *Verrucomicrobiota*. We are currently investigating its reproducibility by focusing on various microbial sources from environmental samples.

## Citation

Morishita, Y., Iwashita, T., Kanno, M., Tamaki, H., Kamagata, Y., Toyama, T., et al. (2025) Improvements in the Duckweed-Microbe Co-cultivation Method for the Stable and Efficient Isolation of Rarely Cultivated Bacteria Using Microfilter Membranes. *Microbes Environ ***40**: ME24075.

https://doi.org/10.1264/jsme2.ME24075

## Supplementary Material

Supplementary Material

## Figures and Tables

**Fig. 1.**
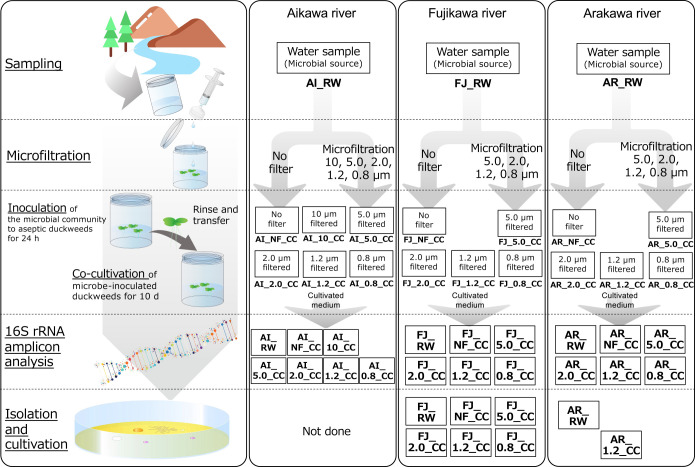
Schematic image for constructing the “duckweed-microbe co-cultivation system” and sample information used in this study. Sample IDs are shown in bold type.

**Fig. 2.**
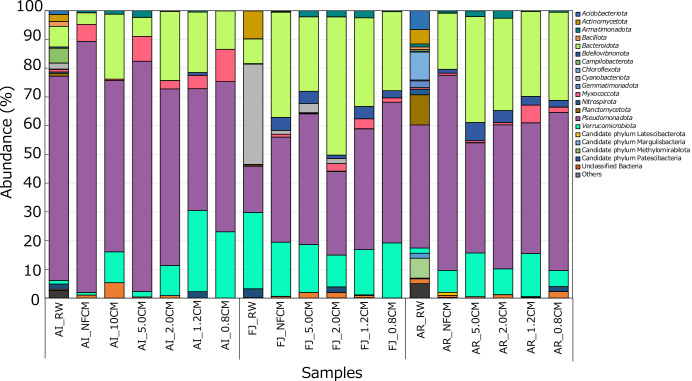
Microbial compositions in samples from “duckweed-microbe co-cultivation systems” and three river waters at the phylum level. Sequences of taxa with a maximum abundance <1.0% in each sample were assembled as “Others”.

**Fig. 3.**
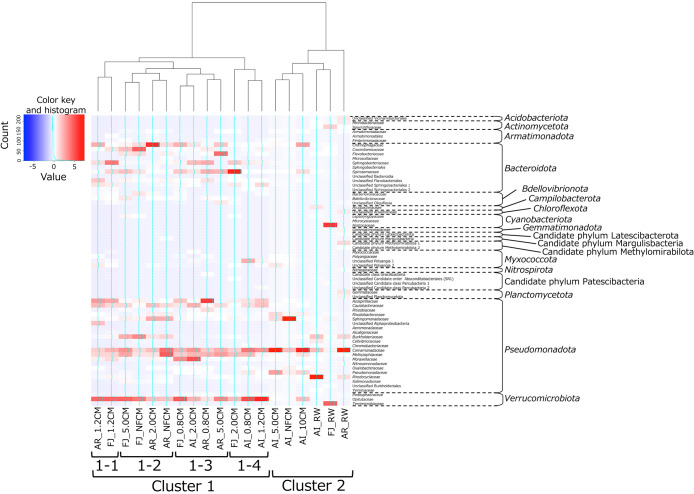
Heat map showing the distribution of bacterial families in samples from co-cultivation systems and their microbial sources. Taxa with a maximum abundance <1.0% in each sample were not included.

**Table 1.** Phylogenetic classification of isolates based on 16S rRNA gene sequences

RFLP group	Representative strain	No. of isolates								Closest authentic species (Accession No.)	Phylum (Class)	Similarity (%)	Compared length (bp)
		FJ_RW	FJ_NFCM	FJ_5.0CM	FJ_2.0CM	FJ_1.2CM	FJ_0.8CM	AR_RW	AR_1.2CM				
1	MRST-41					1	2			*Novosphingobium taihuense* (AY500142)	*Pseudomonadota* (Alpha)	99.02	716
2	MRST-32					2	6			*Caulobacter segnis* (AB023427)	*Pseudomonadota* (Alpha)	100	652
**3**	**MRST-37**						**1**			***Niveispirillum fermenti*** ** (JX843283)**	***Pseudomonadota*** ** (Alpha)**	**97.23**	**713**
**4**	**MRST-71**					**3**	**3**			***Ramlibacter agri*** ** (MN685325)**	***Pseudomonadota*** ** (Beta)**	**97.78**	**812**
**5**	**MRST-20**				**2**	**3**	**5**			***Pedobacter xinjiangensis*** ** (EU734803)**	***Bacteroidota***	**93.88**	**815**
6	MRST-56						1			*Aquincola amnicola* (LN794224)	*Pseudomonadota* (Beta)	99.88	818
**7**	**MRST-25**						**2**			***Azorhizobium caulinodans*** ** (D11342)**	***Pseudomonadota*** ** (Alpha)**	**98.55**	**760**
8	MRST-61			1		6	2	3		*Methylophilus methylotrophus* (AB193724)	*Pseudomonadota* (Beta)	98.9	818
9	MRST-26					1	2			*Azospirillum thiophilum* (EU678791)	*Pseudomonadota* (Alpha)	99.58	716
10	MRST-18						1			*Parasediminibacterium paludis* (MT760284)	*Bacteroidota*	98.84	782
11	MRST-27						1			*Bosea massiliensis* (AF288309)	*Pseudomonadota* (Alpha)	99.84	635
12	MRST-13						2			*Fluviicola kyonggii* (KY117481)	*Bacteroidota*	98.79	822
13	MRST-35					1	1			*Ferrovibrio denitrificans* (GQ365620)	*Pseudomonadota* (Alpha)	99.72	766
**14**	**MRC-3**					**2**	**2**			***Rariglobus hedericola*** ** (MN197844)**	***Verrucomicrobiota***	**91.82**	**805**
15	MRST-55					1				*Aquabacterium soli* (MN911385)	*Pseudomonadota* (Beta)	99.38	808
**16**	**MRST-58**				**1**				**1**	***Curvibacter delicatus*** ** (AF078757)**	***Pseudomonadota*** ** (Beta)**	**98.14**	**807**
17	MRST-40			2		1				*Novosphingobium subterraneum* (AB025014)	*Pseudomonadota* (Alpha)	100	741
**18**	**MRST-7**					**1**				***Emticicia agri*** ** (LC434627)**	***Bacteroidota***	**91.46**	**759**
19	MRST-48					2				*Rhizorhabdus phycosphaerae* (MT482605)	*Pseudomonadota* (Alpha)	99.87	774
20	MRST-76					1				*Lacunisphaera anatis* (KX058883)	*Verrucomicrobiota*	100	792
21	MRST-67					3				*Methylophilus quaylei* (AY772089)	*Pseudomonadota* (Beta)	99.18	747
**22**	**MRC-2**								**2**	***Horticoccus luteus*** ** (MW663766)**	***Verrucomicrobiota***	**92.28**	**777**
23	MRST-47					1				*Rhizobium rosettiformans* (EU781656)	*Pseudomonadota* (Alpha)	99.6	743
24	MRST-2	1								*Microcella frigidaquae* (EF373534)	*Actinomycetota*	99.33	747
**25**	**MRST-10**	**4**								***Flavobacterium cheonhonense*** ** (GU295972)**	***Bacteroidota***	**97.47**	**794**
26	MRST-9	1								*Flavobacterium cheniae* (AB682144)	*Bacteroidota*	100	783
27	MRST-73	5								*Vogesella lacus* (EU287927)	*Pseudomonadota* (Beta)	100	791
28	MRST-5	1								*Arcicella rigui* (HM357635)	*Bacteroidota*	99.6	760
29	MRST-34	2	2							*Erythrobacter tepidarius* (AB033328)	*Pseudomonadota* (Alpha)	99.61	778
30	MRST-57						1			*Curvibacter delicatus* (AF078757)	*Pseudomonadota* (Beta)	99.75	798
**31**	**MRST-52**						**1**			***Shinella yambaruensis*** ** (AB285481)**	***Pseudomonadota*** ** (Alpha)**	**97.63**	**676**
**32**	**MRST-44**					**1**	**2**			***Phenylobacterium panacis*** ** (KT191026)**	***Pseudomonadota*** ** (Alpha)**	**98.54**	**752**
33	MRST-30					1				*Caulobacter fusiformis* (AJ227759)	*Pseudomonadota* (Alpha)	98.77	733
**34**	**MRC-5**					**3**				***Oleiharenicola lentus*** ** (MH493679)**	***Verrucomicrobiota***	**96.59**	**821**
**35**	**MRST-14**						**1**			***Fluviicola kyonggii*** ** (KY117481)**	***Bacteroidota***	**97.75**	**786**
36	MRST-22					1				*Alteraurantiacibacter buctensis* (KJ599648)	*Pseudomonadota* (Alpha)	99.46	734
37	MRST-51				4					*Sandarakinorhabdus cyanobacteriorum* (MG519281)	*Pseudomonadota* (Alpha)	100	766
38	MRST-65				7				1	*Methylophilus methylotrophus* (AB193724)	*Pseudomonadota* (Beta)	99.63	811
**39**	**MRC-8**				**2**	**1**				***Rariglobus hedericola*** ** (MN197844)**	***Verrucomicrobiota***	**93.21**	**785**
**40**	**MRST-46**				**1**					***Rhizobium azibense*** ** (JN624691)**	***Pseudomonadota*** ** (Alpha)**	**98.28**	**656**
41	MRST-49				1					*Rhodobacter ruber* (LT852521)	*Pseudomonadota* (Alpha)	99.35	622
42	MRST-29				1					*Caulobacter daechungensis* (JX861096)	*Pseudomonadota* (Alpha)	99.07	773
**43**	**MRST-16**								**1**	***Paraflavitalea soli*** ** (CP032157)**	***Bacteroidota***	**95.64**	**805**
44	MRST-59								2	*Methylophilus leisingeri* (AB193725)	*Pseudomonadota* (Beta)	99.27	821
**45**	**MRC-12**								**1**	***Lacunisphaera anatis*** ** (KX058883)**	***Verrucomicrobiota***	**95.82**	**814**
**46**	**MRST-4**								**1**	***Fimbriimonas ginsengisoli*** ** (GQ339893)**	***Armatimonadota***	**91.82**	**793**
**47**	**MRC-10**								**1**	***Horticoccus luteus*** ** (MW663766)**	***Verrucomicrobiota***	**94**	**826**
48	MRST-28								2	*Brevundimonas humi* (KY117472)	*Pseudomonadota* (Alpha)	100	765
**49**	**MRC-9**								**5**	***Rariglobus hedericola*** ** (MN197844)**	***Verrucomicrobiota***	**93.21**	**824**
50	MRST-8							2		*Emticicia aquatica* (KP765737)	*Bacteroidota*	100	798
**51**	**MRST-43**							**1**		***Phenylobacterium muchangponense*** ** (HM047736)**	***Pseudomonadota*** ** (Alpha)**	**98.34**	**664**
52	MRST-74							3		*Acinetobacter brisouii* (DQ832256)	*Pseudomonadota* (Gamma)	99.5	798
53	MRST-31							3		*Caulobacter henricii* (AJ227758)	*Pseudomonadota* (Alpha)	99.44	758
**54**	**MRST-23**							**2**		***Asticcacaulis biprosthecium*** ** (AJ247193)**	***Pseudomonadota*** ** (Alpha)**	**97.91**	**670**
55	MRST-3							1		*Mycolicibacterium anyangense* (KJ855063)	*Actinomycetota*	99.47	756
**56**	**MRST-50**								**1**	***Rhodopseudomonas thermotolerans*** ** (LC221830)**	***Pseudomonadota*** ** (Alpha)**	**97.76**	**753**
57	MRST-62								1	*Methylophilus methylotrophus* (AB193724)	*Pseudomonadota* (Beta)	99.14	695
58	MRST-63				2					*Methylophilus methylotrophus* (AB193724)	*Pseudomonadota* (Beta)	98.74	794
**59**	**MRST-69**				**9**	**1**		**1**	**2**	***Ramlibacter aquaticus*** ** (MW138094)**	***Pseudomonadota*** ** (Beta)**	**98.15**	**822**
**60**	**MRST-75**								**1**	***Piscinibacter gummiphilus*** ** (AB609313)**	***Pseudomonadota*** ** (Gamma)**	**98.37**	**797**
61	MRST-66				1					*Methylophilus methylotrophus* (AB193724)	*Pseudomonadota* (Beta)	98.97	773
62	MRST-39		12							*Novosphingobium ginsenosidimutans* (JQ349046)	*Pseudomonadota* (Alpha)	99.25	675
63	MRST-60		2							*Methylophilus luteus* (FJ872109)	*Pseudomonadota* (Beta)	99.87	806
**64**	**MRST-11**		**1**							***Flavobacterium cheonhonense*** ** (GU295972)**	***Bacteroidota***	**97.75**	**755**
65	MRST-21		1							*Sediminibacterium goheungense* (JN674641)	*Bacteroidota*	100	796
66	MRST-64			5						*Methylophilus methylotrophus* (AB193724)	*Pseudomonadota* (Beta)	98.84	777
67	MRST-24		2	2						*Asticcacaulis excentricus* (AB016610)	*Pseudomonadota* (Alpha)	100	730
68	MRST-42		1							*Phenylobacterium conjunctum* (AJ227767)	*Pseudomonadota* (Alpha)	99.74	756
**69**	**MRST-68**			**3**						***Ottowia flava*** ** (MH790863)**	***Pseudomonadota*** ** (Beta)**	**97.67**	**643**
70	MRST-15			2						*Lacibacter daechungensis* (KC759435)	*Bacteroidota*	99.48	789
**71**	**MRST-38**			**1**						***Niveispirillum fermenti*** ** (JX843283)**	***Pseudomonadota*** ** (Alpha)**	**97.94**	**687**
72	MRST-36			1						*Neorhizobium alkalisoli* (EU074168)	*Pseudomonadota* (Alpha)	99.18	780
73	MRST-53			3						*Xanthobacter flavus* (X94199)	*Pseudomonadota* (Alpha)	99.2	624
74	MRST-33			2						*Chakrabartia godavariana* (MF083694)	*Pseudomonadota* (Alpha)	100	618
**75**	**MRST-70**			**1**						***Ramlibacter monticola*** ** (KY313410)**	***Pseudomonadota*** ** (Beta)**	**98.27**	**637**
76	MRST-72		2							*Sphaerotilus montanus* (EU636006)	*Pseudomonadota* (Beta)	99	699
77	MRST-45			1						*Phreatobacter oligotrophus* (HE616165)	*Pseudomonadota* (Alpha)	100	726
**78**	**MRC-7**					**1**				***Horticoccus luteus*** ** (MW663766)**	***Verrucomicrobiota***	**91.77**	**790**
**79**	**MRST-19**			**1**						***Parasediminibacterium paludis*** ** (MT760284)**	***Bacteroidota***	**97.99**	**798**
**80**	**MRST-12**								**1**	***Fluviicola chungangensis*** ** (MH368763)**	***Bacteroidota***	**98.48**	**788**
81	MRST-54								1	*Xanthobacter flavus* (X94199)	*Pseudomonadota* (Alpha)	99.05	742
**82**	**MRST-17**								**1**	***Paraflavitalea soli*** ** (CP032157)**	***Bacteroidota***	**95.27**	**719**
**83**	**MRST-6**						**1**			***Daejeonella rubra*** ** (HQ882803)**	***Bacteroidota***	**93.97**	**791**
**84**	**MRC-1**								**1**	***Polyangium aurulentum*** ** (MK226202)**	***Myxococcota***	**84.53**	**790**
Total		14	23	25	31	38	37	16	26				
*Verrucomicrobiota* strain		0	0	0	2	8	2	0	9				

RFLP groups in which 16S rRNA gene sequences showed less than 98.7% identity with those from authentic species are shown in bold type.
