# Supplementary material for: Improvements in the Duckweed-Microbe Co-cultivation Method for the Stable and Efficient Isolation of Rarely Cultivated Bacteria Using Microfilter Membranes
Source: Microbes Environ. 2025 Aug 20;40(3):ME24075. doi: 10.1264/jsme2.ME24075 (PMC12501865; doi:10.1264/jsme2.ME24075)
Supplement: Supplementary file 1 — Supplementary Material [file 40_24075_s1.pdf]

Supplementary Materials

Improvements in the Duckweed-Microbe Co-cultivation Method for the Stable and Efficient Isolation of Rarely Cultivated Bacteria Using Microfilter Membranes

YOSUKE MORISHITA, TOMOKI IWASHITA, MANABU KANNO, HIDEYUKI TAMAKI, YOICHI KAMAGATA, TADASHI TOYAMA, KAZUHIRO MORI, MASAAKI MORIKAWA AND YASUHIRO TANAKA

Table S1. Numbers of reads, ASVs, and alpha diversity indices for microbial communities in samples

Sample	Target reads	Non-chimeric reads	ASVs	Chao1 (Richness)	Shannon (Evenness)	Good's coverage of the library (%)
AI_RW	42,105	18,524	308	380	7.82	100
AI_NFCM	41,245	35,053	278	319	7.16	99.98
AI_10CM	61,626	51,269	324	352	7.99	100
AI_5.0CM	48,204	38,997	450	542	8.43	100
AI_2.0CM	49,516	42,127	456	543	8.46	100
AI_1.2CM	54,781	44,954	484	591	8.42	100
AI_0.8CM	51,895	42,830	438	525	8.31	100
FJ_RW	57,702	42,422	355	471	7.80	99.99
FJ_NFCM	60,190	47,464	397	477	8.16	100
FJ_5.0CM	74,411	59,542	514	679	8.58	100
FJ_2.0CM	70,667	54,951	458	624	8.34	100
FJ_1.2CM	71,725	58,230	539	684	8.64	99.99
FJ_0.8CM	65,130	52,254	501	625	8.61	99.99
AR_RW	71,745	32,184	538	798	8.69	100
AR_NFCM	75,411	58,208	425	495	8.36	100
AR_5.0CM	67,766	53,851	476	587	8.56	100
AR_2.0CM	63,928	49,501	407	475	8.35	100
AR_1.2CM	68,271	53,671	546	700	8.74	100
AR_0.8CM	74,305	58,418	487	628	8.51	100

Table S2. Maximum viable cell counts grown on the DTS agar plates

Sample	Maximum viable cell counts (CFU/mL)
FJ_RW	$2.5\times10^{6}\pm3.1\times10^{5}$
FJ_NFCM	$1.9\times10^{6}\pm7.0\times10^{5}$
FJ_5.0CM	$3.3\times10^{6}\pm4.2\times10^{5}$
FJ_2.0CM	$3.2\times10^{6}\pm5.3\times10^{5}$
FJ_1.2CM	$7.1\times10^{6}\pm1.7\times10^{6}$
FJ_0.8CM	$1.3\times10^{7}\pm2.8\times10^{6}$
AR_RW	$3.1\times10^{6}\pm2.3\times10^{5}$
AR_1.2CM	$1.6\times10^{7}\pm1.3\times10^{6}$

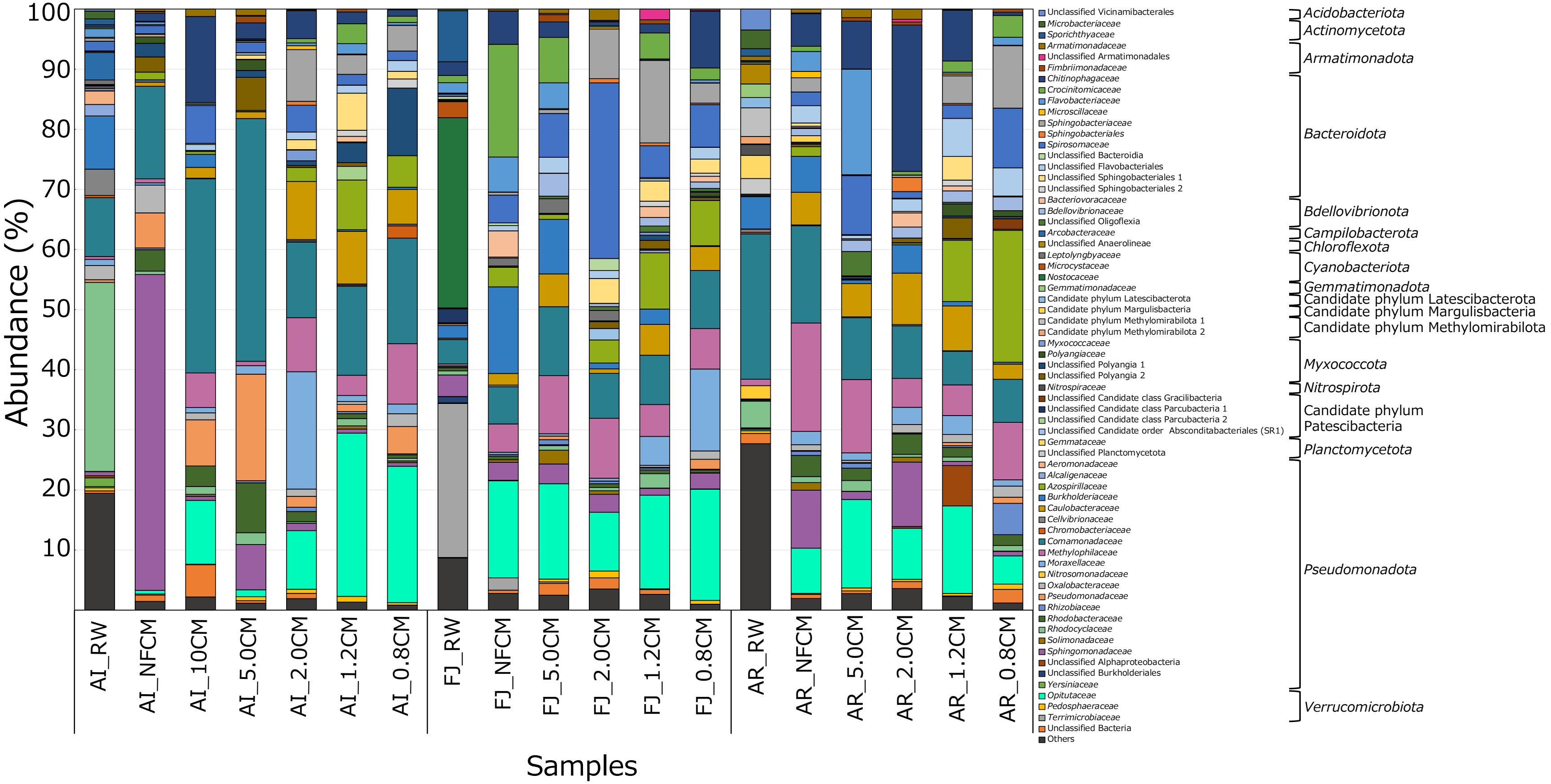

Fig. S1. Microbial compositions in samples from "duckweed-microbe co-cultivation systems" and three river waters at the family level. Sequences of taxa with a maximum abundance < 1.0% in each sample were assembled as “Others”.

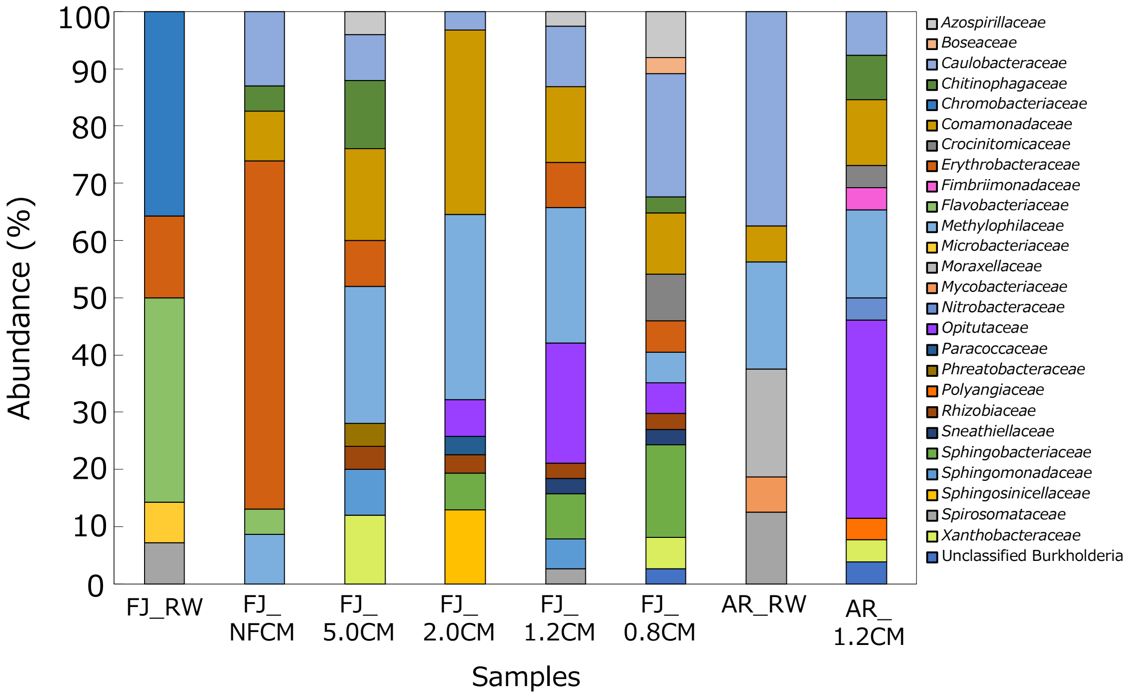

Fig. S2. Taxonomic distribution of isolates at the family level.

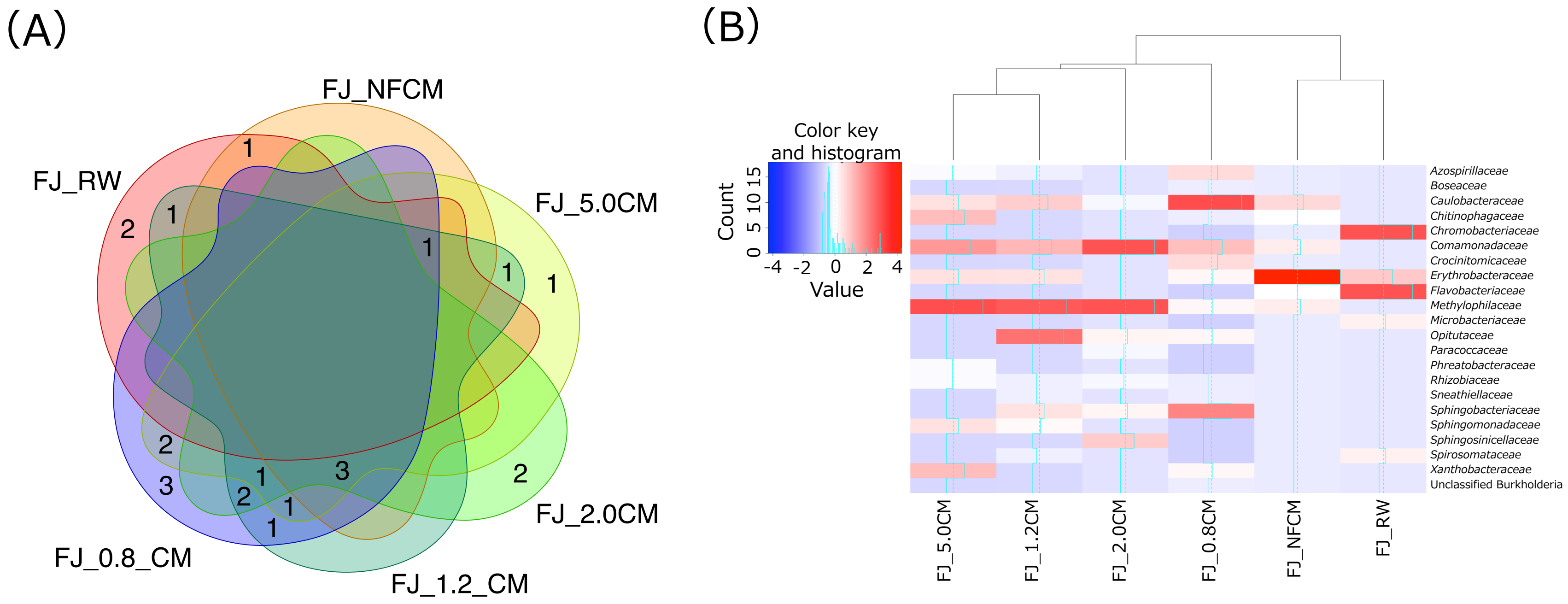

Fig. S3. Taxonomic differences at the family level in isolates from Fujikawa river water-related samples. (A) Venn diagram showing the overlapping of bacterial families. (B) Heat map for the distribution of bacterial families.
